# Congenital Malformations of the Central Nervous System Caused by Bluetongue Virus Serotype 3 (BTV-3) in Two Calves

**DOI:** 10.3390/vetsci12080728

**Published:** 2025-08-01

**Authors:** Phuong Do Duc, Solveig Reeh, Pauline Pöpperl, Tom Schreiner, Natascha Gundling, Andreas Beineke, Peter Wohlsein, Martina Hoedemaker

**Affiliations:** 1 Clinic for Cattle, University of Veterinary Medicine Foundation, 30173 Hannover, Germany; natascha.gundling@tiho-hannover.de (N.G.); martina.hoedemaker@tiho-hannover.de (M.H.); 2 Clinic for Small Animals, University of Veterinary Medicine Foundation, 30559 Hannover, Germany; solveig.brigitta.reeh@tiho-hannover.de; 3 Department of Pathology, University of Veterinary Medicine Foundation, 30559 Hannover, Germanytom.schreiner@tiho-hannover.de (T.S.); andreas.beineke@tiho-hannover.de (A.B.); peter.wohlsein@tiho-hannover.de (P.W.)

**Keywords:** calf, congenital, malformation, bluetongue virus serotype 3, Germany

## Abstract

Since the first emergence of the Bluetongue virus (BTV) in 2006 in Northern Europe, there has been a reported association between BTV Serotype 8 (BTV-8) and brain malformations in calves. The first BTV-3 outbreak in Germany was registered in October 2023. Since then, numbers have increased steadily. In a suckler cow herd in the Lower Saxony region, two Angus calves with clinical signs of diffuse encephalopathy, including ataxia, abnormal gait, and central blindness, were born in autumn 2024. Both calves were submitted for Magnetic Resonance Imaging (MRI) and pathological examination, revealing hydranencephaly and internal hydrocephalus, respectively. BTV-3 was detected in blood and tissue samples of both calves using BTV-specific real-time PCR. The presented findings demonstrate that there seems to be an association between transplacental BTV-3 infections and congenital malformations in calves, as previously reported for BTV-8 and -10.

## Simple Summary

Bluetongue disease is a well-documented viral infection in ruminant wild and domestic animals. The virus is transmitted by midges. Infected animals show symptoms ranging from fever, bloody nasal discharge, oral lesions, lameness, decrease in milk yield, abortion, and stillbirth to the characteristic blue coloring of the tongue. This virus can also be transmitted through the placenta during pregnancy, causing brain deformations in the surviving fetuses. Live-born calves subsequently show weakness, blindness, and an uncoordinated gait and are often referred to as “dummy calves”. Brain malformations in two infected calves were diagnosed using Magnetic Resonance Imaging examination, which was verified via pathological examination. This finding confirms the ability of the Bluetongue virus to cause such brain deformations.

## 1. Introduction

Congenital brain malformation caused by fetal intrauterine viral infection is well-documented in calves and is often associated with dystocia, increased mortality, and economic burden [1,2]. Malformations occur most commonly in the central nervous system (CNS), including hydranencephaly, internal hydrocephalus, porencephaly, cerebellar hypoplasia, and micromyelia, but they also occur in the musculoskeletal system [3]. Hydranencephaly is defined as severe, bilaterally symmetric cerebral necrosis with replacement of neuroparenchymal hemispheres with cerebrospinal fluid (CSF), lined by surviving, variably thick and transparent brain parenchyma and the overlying, intact leptomeninges [4]. Internal hydrocephalus is a common structural defect of the CNS of cattle and is caused by genetic factors [5]. It is defined as a dilation of all or part of the ventricular system in the brain [4]. Hydrocephalus is categorized according to different criteria. The first one is the communicating hydrocephalus, which is uncommon and involves enlargement of the ventricular system without detectable obstructive lesions [4]. The second type, termed non-communicating hydrocephalus, results from complete or partial obstruction of the intra- and extraventricular pathways, leading to abnormal CSF flow. The increased intraventricular pressure leads to dilation of the intraventricular spaces and possibly the spinal canal (hydromyelia) [4]. The third form is the compensatory or ex vacuo hydrocephalus. It develops following the intrauterine or postnatal loss of neuroparenchyma and is characterized by normal intraventricular pressure independent of any change in obstruction or alteration in CSF production [4,6]. In contrast, external hydrocephalus refers to the expansion of the extraventricular subarachnoid space [6]. Of note, the term porencephaly refers to the formation of a focal cyst of variable size within the cerebral hemispheres, lined by normal brain tissue and filled with CSF. All the aforementioned malformations can present either simultaneously or individually [7].

Hydranencephaly, internal hydrocephalus, and ex vacuo hydrocephalus in cattle can be caused by teratogenic and neurotrophic viruses such as Akabane virus (AKAV), Rift Valley fever virus (RVFV), Border disease virus (BDV), Cache Valley virus (CVV) [8], Aino virus (AV) [3], Bovine viral diarrhea virus (BVDV), Schmallenberg virus (SBV) [9,10], and Bluetongue virus (BTV) [3,11]. BTV causes an infectious but noncontagious disease and is classified into the family *Sedoreoviridae* and genus *Orbivirus*. Orbiviruses can be divided into a number of serotypes based on neutralization of the outer capsid proteins by specific neutralizing antibodies [12]. To date, 28 serotypes of BTV have been identified, differing in antigenicity, genome sequence, and geographic distribution [12,13,14,15,16,17,18]. BTV is transmitted by biting midges of the family *Ceratopogonidae* and genus *Culicoides* [19] and significantly affects domestic and wild ruminants, causing an economically important disease [1,2,20]. Usually, BTV primarily proliferates in endothelial cells, causing cell damage and necrosis. This vascular injury leads to thrombosis, tissue infarction, and hemorrhage [21,22]. Clinical signs range from fever, bloody nasal discharge, and oral lesions to characteristic cyanosis, respiratory distress, lameness with coronitis, progressive debility, and death [23].

An association between hydranencephaly and BTV infection during early pregnancy in cattle has been demonstrated experimentally, using purified BTV-10 [24] and naturally occurring BTV-8 [25,26,27,28]. In 2006, the emergence of BTV-8 in Europe was reported, and the transplacental transmission of BTV along with its link to congenital brain defects was confirmed [25,29,30]. However, the cerebral malformations only occurred due to BTV following transplacental transmission with BTV [31], and the severity of the outcome depends on the gestational stage at the time of infection [32]. The precursors of neurons and glial cells of up to approximately 130 days of gestation are particularly susceptible and vulnerable, and an infection with BTV results in widespread cellular necrosis [33]. Surviving fetuses infected prior to mid-gestation are often born with severe CNS malformations, ranging from hydranencephaly to cerebellar defects [34]. In contrast, fetal infection at a later stage of gestation leads to cerebral cysts and dilated ventricles, while infection occurring a few weeks prior to birth results in mild encephalitis with no gross brain malformation [24,31,35]. Consequently, the outcome of a BTV infection is dependent on the developmental stage and immunocompetence of the conceptus [29,31,33]. After a BTV-free period of several years, BTV-3 surprisingly emerged in the Netherlands in September 2023 for the first time and spread rapidly. By October 2023, the Friedrich-Loeffler-Institute confirmed the first BTV-3-associated infections in Germany [36]. Given the virus’s prior detection in the Netherlands and its rapid spread, these cases were not entirely unexpected. Concurrent outbreaks were also reported in Belgium and the United Kingdom [37].

This report describes the clinical, MRI, virological, and pathological findings collected at the University of Veterinary Medicine Hannover, Foundation, in two calves born in Germany in November and December 2024 on the same farm by dams infected with BTV-3 during gestation.

## 2. Case Description

Both reported calves were Aberdeen Angus, born in due time without complications, and originated from a suckler cow herd. Calf 1 was born on the 12 December, and Calf 2 was born on the 8 November 2024. Their dams were later confirmed to be BTV antibody-positive by ELISA in February 2025. At the suspected time of infection, specific vaccines against BTV-3 were not yet available; thus, the dams were not protected. The dead bodies of both calves were submitted for examination in December 2024.

### 2.1. Calf 1

According to a statement from the farmer, Calf 1 was born without complications. Immediately after birth, the calf displayed disorientation upon movement, was unable to stand and suckle on its own, and exhibited abnormal posture (hyperextension of the legs and kyphosis) and an uncoordinated gait. The calf died within a few hours of birth, limiting the availability of clinical data. The calf was submitted for MRI immediately after death and subsequent pathologic examination.

### 2.2. Calf 2

**Figure 1:**
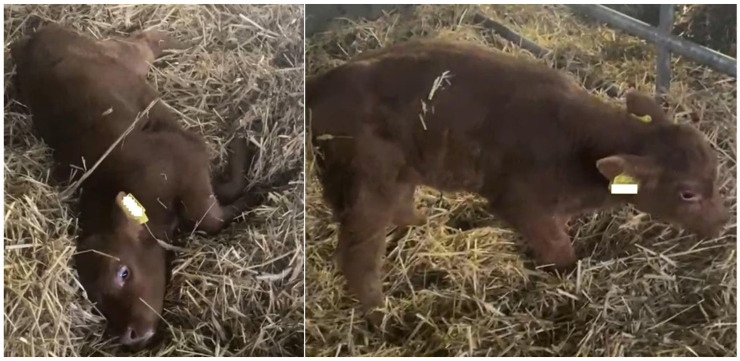
Five-week-old, female, transplacental-infected BTV-3-positive calf showing severe CNS dysfunction; spontaneous falling down and stargazing (**left**); and standing up with assistance and walking with an uncoordinated gait (**right**). The calvarium is normal-sized.

Following an unremarkable birth, Calf 2 exhibited neurological abnormalities including an uncoordinated gait, episodes of falling, stargazing (cervical hyperextension more than 90°), and fluctuating levels of consciousness. Blindness was suspected, as the calf showed difficulty locating and suckling its dam. Although the calf was barely able to stand in a coordinated manner, it was capable of sucking on its own and was bottle-fed (Figure 1). Despite intensive care, there was no demonstrable clinical improvement. In light of the findings upon examination of Calf 1, the decision to euthanize Calf 2 was made at 6 weeks by the owner and performed by their veterinarian. The body was then handed over to the University of Veterinary Medicine Hannover, Foundation, for further examination.

## 3. Results

### 3.1. General Clinical Findings (Calf 2)

General clinical examination revealed age-appropriate physical development, with good nutritional and grooming status. However, overall, the calf’s condition was reduced; Calf 2 was recumbent in lateral position. Vital parameters were within normal limits. Urination and defecation were undisturbed.

### 3.2. Neurological Findings (Calf 2)

Neurological examination demonstrated fluctuating levels of consciousness ranging from apathy to stupor. The calf exhibited intermittent opisthotonus and generalized tremors. In the head and trunk, jerk-like myoclonic movements were observed. The calf was minimally ambulatory, displaying tetraparesis and generalized mixed ataxia. It had an impaired ability to stand and walk, with occasional episodes of collapse.

Cranial nerve examination demonstrated absent menace reflexes and bilateral vision. Pupillary light reflexes remained intact, suggesting central blindness. A bilateral ventrolateral strabismus was noted, and the palpebral reflex was reduced on the right side. All the other findings in the neurological examination were unremarkable and within normal limits.

Based on the clinical findings, a diffuse encephalopathy was suspected. Differential diagnoses included congenital anomalies, infectious etiologies, or metabolic causes.

### 3.3. MRI Findings

A postmortem MRI scan of the skull was performed using a 3.0 Tesla MRI scanner (Achieva, Philips Medical Systems, Best, The Netherlands), followed by gross and histopathological examination. MRI sequences included transverse, sagittal, and dorsal T2-weighted images; transverse Fluid Attenuated Inversion Recovery (FLAIR); sagittal, transverse, and dorsal T1-weighted images without contrast; and gradient echo and diffusion-weighted sequences.

**Figure 2:**
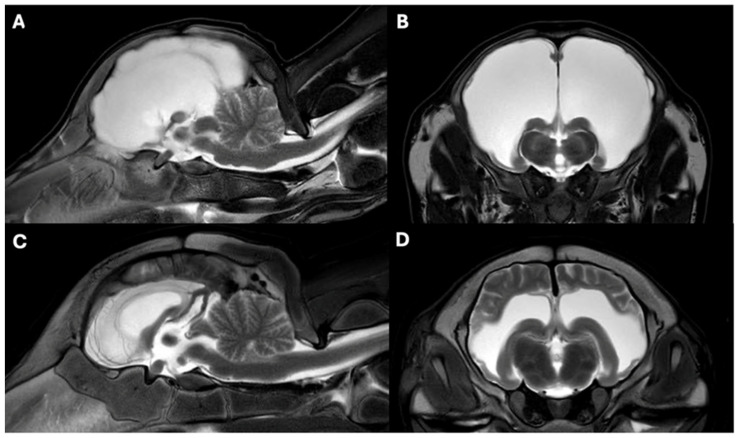
MRI findings in T2-weighted midsagittal (**A**,**C**) and transverse (**B**,**D**) images of Case 1 (**C**,**D**) and Case 2 (**A**,**B**). In Case 2 (**A**,**B**), hydranencephaly is suspected due to the absence of cerebral parenchyma in the cerebral hemispheres, which is replaced by CSF. In Case 1 (**C**,**D**), an internal hydrocephalus is suspected. The lateral ventricles are markedly dilated, but the surrounding parenchyma is still visible.

In Case 1, there was a moderate dilatation of the lateral ventricles. Intact brain parenchyma was still present surrounding the ventricles laterally. The findings were consistent with internal hydrocephalus (Figure 2).

In Case 2, a marked reduction in cerebral parenchyma was observed in the area of the cerebral hemispheres. At the level of the cerebral hemispheres, there was a severe, bilaterally symmetrical intra-axial accumulation of T2. Due to the T2 hyperintense signal in the ventricles, which is suppressed on FLAIR, CSF (white areas) is within the ventricles. The cerebellum and brainstem appeared normal. Based on MRI, a diagnosis of hydranencephaly was established (Figure 2).

### 3.4. Pathomorphological Findings

#### 3.4.1. Case 1

**Figure 3:**
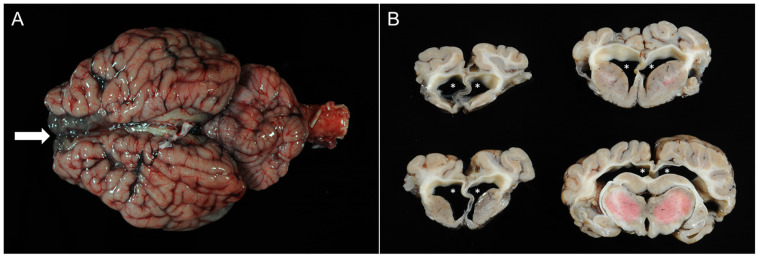
Case 1: calf with internal hydrocephalus. (**A**) Dorsal aspect of the native brain ex situ with translucent frontal lobe (arrow) due to the dilation of lateral ventricles. (**B**) Coronal sections of the formalin-fixed brain. Upper left: rostral frontal lobe; lower left: frontal lobe with basal nuclei; upper right: frontal lobe at the level of corpus callosum and basal nuclei; lower right: diencephalon at the level of the thalamus. The lateral ventricles are bilaterally symmetrically dilated in all sections (asterisks). The central pink coloration of the thalamus (lower right) is caused by incomplete formalin fixation.

The male calf had a body weight of 40 kg with good body condition for its age. Gross examination of the brain showed a moderate internal hydrocephalus with bilateral dilatation of the lateral ventricles and partial loss of the cortical brain tissue (Figure 3). The diencephalon, mesencephalon, and rhombencephalon appeared normal, both grossly and histologically. Histopathologic examination of cortical brain tissue revealed multifocal mild lympho-histiocytic meningoencephalitis, multifocal areas of necrosis and dystrophic mineralization, and high-grade hypomyelination of the white matter.

Within the liver, mild portal and partially centrolobular fibrosis was observed, as well as mild bile duct proliferations with the presence of multiple arterioles. Additional findings included marked cortical hemorrhages with hyaline microthrombi in the adrenal glands as well as alveolar edema and emphysema of the lungs.

#### 3.4.2. Case 2

**Figure 4:**
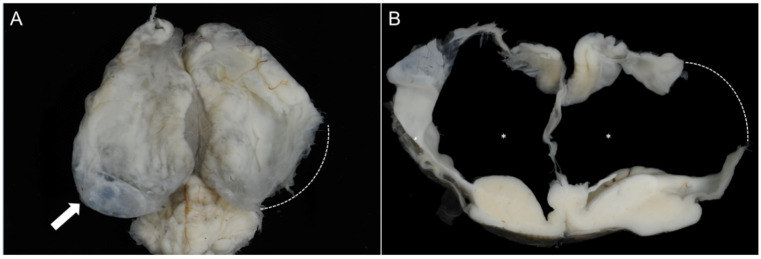
Case 2: calf with hydranencephaly. (**A**) Dorsal aspect of the formalin-fixed brain. The hemispheres are partially collapsed, and the thin residual cortical tissue shows a focal translucent area (arrow), indicating a fluid-filled lateral ventricle. (**B**) Coronal section at the level of corpus callosum and basal nuclei with severe loss of cortical brain parenchyma and bilateral symmetrically highly dilated lateral ventricles (asterisks). The disconnected lining of the ventricle is an artifact (stippled line) caused by the removal of the brain from the cranial cavity.

The second case was a female calf with a body weight of 42 kg. Examination of the brain revealed hydranencephaly characterized by subtotal loss of cortical hemispheres and replacement by fluid-filled ventricular cavities, lined by fragile remnants of brain parenchyma and the intact meninges (Figure 4). As with Case 1, the diencephalon, mesencephalon, and rhombencephalon did not show any abnormalities.

Histologically, a multifocal mild perivascular lympho-histiocytic infiltration was found in the remaining cortical brain tissue.

Additionally, the thymus was well-developed and showed multifocal acute hemorrhages. There was mild congestion noted in the spleen and liver. The lungs showed mild, diffuse, acute alveolar edema.

### 3.5. Virological Findings

BTV-3 infection was confirmed in both calves by specific reverse transcriptase quantitative PCR using the test virotype BTV pan/3 2.0 RT-PCR Kit (Indical Bioscience (Leipzig, Germany), FLI-C 158). In Calf 1, BTV RNA was detected in spleen tissue: BTV-3 gene fragment-positive (Ct-value: 30.11) and BTVPAN gene fragment-positive (Ct-value: 26.61). In Calf 2, BTV-3 infection was confirmed in EDTA blood: BTV-3 gene fragment-positive (Ct-value: 29.27) and BTVPAN gene fragment-positive (Ct-value: 26.17). For both calves, BVDV infection was ruled out by PCR (virotype BVDV RT-PCR Kit, Indical Bioscience, FLI-B 451) from organ samples tested at the Veterinary Institute Hannover. Immunohistochemistry on representative tissue sections was performed as previously described, using a specific antiserum against SBV N protein in a dilution of 1:3000 and the EnVision detection system [10]. In both calves, immunohistochemistry for the SBV N protein was negative.

## 4. Discussion

Our findings relate the neurological deficiencies to fetal infections with BTV described in other studies [26,38]. Both calves exhibited central nervous signs that correspond to severe malformations of the brain. Etiological investigations revealed intrauterine infections with BTV-3 as the underlying cause of the disease.

Briefly, brain parenchyma as well as the meninges of both calves showed inflammatory changes and necrosis consistent with intrauterine viral infection. In Case 1, these findings were interpreted as internal hydrocephalus, and in Case 2, hydranencephaly was diagnosed. BTV-3-specific genome fragments were detected in samples from both calves. Infections with BVDV and SBV as possible etiological differential diagnoses causing congenital CNS malformations were ruled out in both cases by PCR and immunohistochemistry, respectively.

Previous studies have demonstrated the possibility of BTV crossing the placental barrier and causing congenital malformations in sheep [39,40] and in cattle by BTV-8 [41,42]. In the studies involving BTV-10 and BTV-13, the virus could not be reisolated in organ tissue or blood samples from the live-born calves with brain defects [24,43], considering that only cell culture was available at that time; thus, virus detection techniques were limited. In more recent studies, BTV-8 was found using RT-PCR in spleen tissue and in blood samples [25,28,44]. The RT-PCR-positive results of the spleen tissue and the blood samples from the described calves support the concept that BTV-3 is able to cross the placenta and infect the fetus. Darpel et al. [22] reported a high rate of transplacental transmission rates (22–47%), and the likelihood of this transfer increased later in gestation, making the dam become infected. However, in their study, Zanella et al. [44] found that 16% of BTV-8-positive calves were born to the RT-PCR-positive dams, which is comparable to the 18.5% BTV-8-positive fetuses reported by De Clercq et al. [41]. Moreover, according to van den Brink et al. [37], BTV-3 seems to spread faster and cause a more severe impact than, e.g., BTV-8. Given our findings in two calves with central nervous malformations and confirmation of BTV-3 infections, this raises the possibility that BTV-3 may have a higher likelihood of transplacental infections of the fetus than other serotypes.

The neurological signs, such as uncontrolled gait, disbalance, and blindness or weakness, were found in both calves. These behavioral abnormalities were already described in association with BTV [22,26,28]. The teratogenic effect of BTV on cattle is well established, with congenital abnormalities such as the detected hydrocephalus in Calf 1 and the hydranencephaly in Calf 2 being repeatedly reported [24,25,26,28,38]. The severity of the developmental brain defects depends on the gestation stage at the time of infection as well as the BTV serotypes involved [43]. In our report, postmortem examination revealed normal calvaria and a necrotizing encephalopathy in both calves. In Case 1, the frontal lobe was translucent due to the dilatation of lateral ventricles, and in Case 2, the cerebral hemispheres were filled with CSF. The residual cortical tissue was thin and focally transparent. The pathological findings in these calves satisfy the criteria for hydrocephalus in Case 1 and for hydranencephaly in Case 2 [4,6]. Although congenital malformations can potentially be caused by other teratogenic viruses like AKAB, AV, CVV, or RVFV during pregnancy, no infections of this nature have been detected in Germany. Although SBV was detected in Germany in 2011 [45] and in Belgium in 2012 [46], there are presently no reports of active infections. Moreover, none of the examined calves showed any signs of musculoskeletal deformation associated with SBV [46], and it was, furthermore, ruled out by testing. Thus, an association between these viruses and the described encephalopathies is unlikely. Additionally, only BTV is topically present in Europe. Moreover, BVDV was ruled out by PCR examination in the presented cases. Furthermore, since the re-emergence of BTV in Europe, only BTV-3 has been identified in Germany, with an increase in the number of infections reported through December 2024. Considering these findings and the BTV-3-positve testing in both calves, BTV-3 is the most likely cause for the described congenital malformations.

This is the first report of BTV-3-associated malformations diagnosed using MRI. MRI images and pathological findings are consistent with brain malformations caused by intrauterine infections with BTV, both naturally and experimentally.

Based on the observations in the presented two cases, it can be concluded that BTV-3 infection can have a significant impact on the health of calves. However, to date, there have been no additional studies published describing transplacental BTV-3 infections in cattle, either under experimental or natural conditions. The presented findings demonstrate and support the idea that BTV-3 can cross the placenta and cause fetal brain malformations, which represent an additional economic burden to affected herds. Furthermore, BTV-3 seems to be highly virulent, which deems vaccination protocols necessary and essential to protect animal health and welfare and reduce financial loss.

Since the start of the availability of a vaccine specifically against BTV-3 near the end of 2024, country-wide vaccination protocols have been encouraged in Germany. Two vaccinations, 3 weeks apart, are suggested. The end of winter is the ideal timing to create an effective immune response before the emergence of midges.

## 5. Conclusions

On a final note, our findings suggest the potential of BTV-3 to cross the ruminant placental barrier and infect the fetus. Surviving fetuses presented with neurological signs ranging from incoordination and fluctuating levels of consciousness to blindness and weakness, which are associated with congenital malformations like internal hydrocephalus or hydranencephaly. However, such calves can live, needing intensive care.

## Data Availability

The raw data supporting the conclusions of this article can be requested from the corresponding author.
